# Supplementary material for: Maternal prenatal stress induces sex-dependent changes in tRNA fragment families and cholinergic pathways in newborns
Source: Mol Psychiatry. 2025 Apr 5;30(9):4307–19. doi: 10.1038/s41380-025-03011-2 (PMC12339370; doi:10.1038/s41380-025-03011-2)
Supplement: Supplementary file 1 — Supplementary Information [file 41380_2025_3011_MOESM1_ESM.pdf]

Maternal prenatal stress induces sex-dependent changes in tRNA fragment families and cholinergic pathways in newborns

Supplementary Information

Supplementary methods 2
Participant criteria and handling 2
Cholinesterase activity measurements and calculation 2
Serum RNA size selection for sequencing 3
Immune-related measurements.. 4
C-reactive protein (CRP) measurements 4
Umbilical cord whole blood RNA qPCR 4
Assignment of tRF families 5
Converting between tRF annotations 6
Dual Luciferase assay.. 6
Supplementary results 7
Newborns show sex-specific elevation in immune mRNA biomarkers.. 7
Gene Ontology (GO) shows tRF targets spanning transcription regulation pathways 8
MT-Gly-i-tRF family correlates with maternal BMI.. 8
Supplementary Figures.. 10
Supplementary References 26

SUPPLEMENTARY METHODS

Participant criteria and handling

Participant matched exclusion criteria from previous work of the FELICITy cohort [1], mainly, women how suffered from maternal severe illness during pregnancy [2], fetal restricted growth due to serious placental alterations [3], fetal malformations, and/or maternal drug or alcohol abuse cases were excluded from the study. Further, for the serum cholinesterase measurements with Ellman's assay, maternal and newborns samples were matched for relevant dyads and those were randomized to prevent batch effects between stress-by-sex groups in the initial experiment phases. In the RNA extraction the samples were randomized as well, but separately for newborns and mothers' samples, as the extraction protocols were done separately as well.

Cholinesterase activity measurements and calculation

Cholinesterase activity was measured using Ellman's assay [4] in serum samples from 70 mothers and newborns. We used only those who experienced non-emergency vaginal birth, to prevent birth types from confounding the results (58.3%, Supplementary Figure 1b). Samples were defrosted rapidly in a water bath, diluted 1:20 in phosphate buffered saline (PBS), and 10 µL were mixed with 180 µL Ellman's solution in 96 flat-well plates. All samples were measured in triplicate and under three conditions: without inhibitors, with the BChE inhibitor tetraisopropylpyrophosphoramide (iso-OMPA; final concentration 50 µM; Sigma, T1505), and with the AChE inhibitor BW284c51 (final concentration 5 µM; Sigma, A9103). Mother-newborn dyads were measured on the same plate and for each sample all three conditions were measured on the same day. To enable comparison between plates each plate included four controls: (1) a random maternal sample, (2) its corresponding newborn sample, (3) a recombinant AChE protein sample, and (4) a recombinant BChE protein sample. The first

two samples served as normalizing controls and the last two as internal controls. The iso-OMPA plates were incubated for 120 minutes covered in aluminum foil, and in all plates 10 µL acetylthiocholine were added to wells immediately before measurement. Hemolysis was seen in 7% of samples of both mothers and newborns, and to avoid its effect on results absorbance was read at 436 nm instead of the standard 412 nm [5]. 21 kinetic cycles of one minute each were read using a Tecan Spark™ 10M plate reader, with all measurements carried out at room temperature. Measurements were retrieved using the SparkControl Magellan 3.1 program and were further analyzed using R [6] as follows:

Average values of "mean OD/minute" units were converted to “nmol substrate hydrolyzed per minute per ml” units using the Beer-Lambert law (A= εlc; molar absorptivity constant ε 2-nitro-5-thiobenzoate= 13,600), multiplied by the serum dilution factor (1:20). Normalization between plates was based on the maternal and newborn control samples described above and was performed separately for maternal and newborn samples. Values were log-transformed to minimize the effect of outliers on the mean. The normalization factor of plate i, denoted f_i, is computed by

$$f_i = \frac{\sum_{j=1}^{n} \log\left(control_j\right)}{n} - \log(control_i),$$

where n is the number of plates. Let $s_{i,k}$ be the activity of the non-control sample k in plate i. The normalized activity, denoted $s'_{i,k}$, is computed by

$$s'_{i,k} = s_{i,k} \cdot e^{f_i}.$$

Serum RNA size selection for sequencing

After library preparation the samples were pooled and size-selected. This was done by separation on 4% agarose gel (Invitrogen, G401004), with a band between 140-200 bp excised. This included inserts between 20 and 80 bp (both 3' and 5' adaptors are 60 bp long). In the

newborn samples the R1 read length was 80, and in maternal samples it was 70, ensuring that in both cases tRF-halfs, 50 bases long, could be detected.

Immune-related measurements

C-reactive protein (CRP) measurements

Women having rupture of membranes during the first stage of labor or showing signs of infection such as fever received a CRP control from plasma upon entry to the delivery room (n=55). 50 of those experienced non-operational vaginal birth and their serum was used for the analysis. In addition, serum samples from the umbilical cord blood after birth of 37 newborns showing clinical signs of infection (aka. fever, low Apgar, maternal fever or maternal high CRP, etc.) served for CRP measurement. 22 of them were included in the analysis, all scoring CRP of 0.1 mg/dl, except for a single one with CRP of 0.5 mg/dl.

Umbilical cord whole blood RNA qPCR

Umbilical cord whole blood from 122 newborns was collected after birth into PAXgene® tubes (catalog no. 762165). Total RNA was extracted using “PAXgene® Blood RNA Kit (catalog no. 762164) according to manufacturer’s instruction, presenting average RIN values of 7.2 and average yield of 310 ng/µL as measured by Bioanalyzer. RNA was frozen at -80oC and was shipped to the Soreq lab on dry ice, where it was stored again at -80oC until analysis.

44 samples from newborns of normal vaginal delivery who were born to mothers scored either as low or high stress in the PSS-10 were chosen for the analysis, to match the sequenced cohort. Long cDNA synthesis and RT-qPCR for mRNAs were done using qScript™ cDNA Synthesis Kit (Quantabio, 95047) and PerfeCTa® SYBR® Green FastMix® (Quantabio, 95072) with human-specific primers (Supplementary table 11). Quantification was done with the CFX384 Touch Real-Time PCR System (Bio-Rad), producing subsequent Cq values with the CFX Maestro software (Bio-Rad v4.1.2433.1219). Data is presented as relative expression (DDCt)

normalized to two housekeeping genes, GAPDH and ACTB, plots and calculations were done in R.

Assignment of tRF families

tRFs share sequence similarities due to their shared tRNA origin and production mechanism [7]. In our study, we used these characteristics as grouping factors, assigning different tRFs that share genome origin (mitochondrial or nuclear), coded amino acid (as referenced by the parental tRNA gene), and cleavage type (5'-tRF, 5-half, i-tRF, 3-half, 3'-tRF) [8], to the same “tRF family". Using the multiple sequence alignment (MSA) algorithm in the msa R package [9] we sought sequence similarities of all tRF families represented in our newborns data which had more than one member (45 out of 51 families). Of those families, 43 enabled us to produce a consensus sequence that coincided with a real tRF, and our data included 42 of these “consensus tRFs” that were 16-27nt long (Supplementary Table 10, Supplementary Figure 9). It is important to note that we did not find a sequence motif that was shared between all tRF families. Furthermore, unlike the nuclear genome that contains multiple tRNA genes for each amino acid, the mitochondrial (MT) genome carries only one tRNA gene for each amino acid, apart from leucine and serine with two each, and each tRNA can encode groups of four closely related codons due to an unmodified uridine at the wobble position among other related mechanisms [10, 11]. As the majority of our data was comprised of those MT tRFs which are coded in the MT-DNA mostly by a single codon, it sufficed to classify tRFs according to the parental tRNA coded amino acid (Supplementary Figure 4a). Even so, we could show that multiple tRF families constructed from nuclear tRNA genes representing different codons showed similar sequences and united expression patterns (Supplementary Figure 6, Figure 4). In addition, length distribution analysis was performed as follows: mean expression levels of each tRF family under each length it spans was calculated for each participant. Next, Kruskal Wallis test was used for each of the tRF families separately, followed

by FDR correction, to compare their mean expression between the stress-by-sex groups. Together, these steps enabled us to assess the differences between specific tRF families rather than individual tRFs.

Converting between tRF annotations

As MINTmap is more sensitive to the identification of MT-tRNA lookalikes in the nuclear genome [12], we used in our family classification the genome origin as indicated by MINTbase [8] instead of the one indicated by the tDRnamer algorithm [13]. Indeed, 40 tRFs were assigned differently between MINTmap and tDRnamer, all from four families that were assigned as MT by tDRnamer and as nuclear MT-lookalikes by MINTmap (families: Nuc-Glu-3'-tRF, Nuc-Glu-i-tRF, Nuc-Ser-3'-tRF, Nuc-Ser-i-tRF). In addition, three of the tRFs identified by MINTmap were not assigned a tDR name by the tDRnamer algorithm (tRF-20-BQ8BB2S1, tRF-21-2FYOB015D, and tRF-22-0DI6BB5LJ), although the rest of their family members were successfuly identified (MT-Gly-3'-tRF). Therefore, we assigned them a tDR name artificially, based on the tDRnamer guidelines (now mtDR-55:76-Gly-TCC-1, mtDR-54:76-Gly-TCC-1, and mtDR-53:76-Gly-TCC-1 accordingly).

Dual Luciferase assay

HEK293T cells (ATTC, CRL-3216) were grown at 37ºC, 5% CO_2, in DMEM (Merck, D5671) supplemented with FCS (10% final concentration, Merck, F7524), L-glutamine (2mM final concentration, Cytiva, SH30034.01) and Penicillin-Streptomycin-Amphotericin (100 units/mL, 0.10 mg/mL, 0.25 µg/mL, final concentrations, respectively, Cytiva, SV30079.01). Cells were mycoplasma free (MycoBlue® Mycoplasma Detector, BioGate, D101-01). For experiments cells were seeded in black clear-bottomed 96-well plates (Greiner, 655090) at 30,000 cells per well in 75 µL medium and 48 hours later were co-transfected with (i) 125 ng psiCHECK™-2 Vector (Promega) containing the 3'UTR of the human BChE gene (ENST00000264381.8) or the

3'UTR of the human IL6 gene (ENST00000258743.10), downstream to the Renilla luciferase gene between XhoI and NotI and (ii) ssRNA oligo of tRF mtDR-36:57-Gly-TCC-1 (CAA UUA ACU AGU UUU GAC AAC) or a scrambled sequence (AUU ACC GAA UUA CGA UCA UUA). 50 nM were used for IL6 and 100nm for BCHE, followed by 24h (IL6) or 48h (BCHE) incubation. Finaly, cells were assayed using the Dual-Glo® Luciferase Assay System (Promega, E2920) per the manufacturer's instructions, with a Tecan Spark™ 10M plate reader. Results are expressed as the ratio of Firefly to Renilla luciferase luminescence for the tRF normalized to scrambled. Four separate experiments, each in duplicate or triplicate, were performed for each 3'UTR construct.

SUPPLEMENTARY RESULTS

Newborns show sex-specific elevation in immune mRNA biomarkers

The cholinergic system controls the immune landscape and is a major regulator of inflammation [14]. Therefore, we further explored the immune landscape of the participants. Serum C-reactive protein (CRP) levels of mothers showing clinical signs of infection during the first birth stage revealed no significant differences between mothers of the four stress-by-sex groups, although a trend was visible towards lower CRP in control mothers of female newborns (Supplementary Figure 2b). A small cohort of newborns also had their umbilical cord blood checked for CRP levels due to clinical signs of infection, although all but one scored the same low CRP level (0.1 mg/dl).

Nevertheless, we tested immune markers in the newborn groups using RT-qPCR tests of six mRNA regulators and inflammatory immune responders (CXCL1, IL-1β, TGF-β, TLR4, TLR7, TNF). This revealed significant elevation of TGF-β levels in female newborns of the stress group compared to controls, suggesting higher regulatory immune response in females, while male newborns of the stress group presented higher TLR4 levels compared both to their controls

and to the female stress group, suggesting stronger inflammatory response (Supplementary Figure 2c)[15].

Gene Ontology (GO) shows tRF targets spanning transcription regulation pathways

GO analysis was preformed using the clusterProfiler R package [16] on the miRDB predicted targets of four marker groups shared by the male and female newborn comparisons: (1) MT-tRFs decreasing in the stress groups (262 tRFs with 8693 targets)s, (2) Nuc-tRFs increasing in the stress groups (158 tRFs with 1857 targets), (3) CholinotRFs (38 tRFs with 5581 targets), and (4) CholinomiRs (16 miRs with 6286 targets) (Supplementary Figure 6a,b). Top pathways of molecular functions revolved around DNA and RNA binding and regulation, with most shared by the decreasing MT-tRFs, CholinotRFs and CholinomiRs target groups, whereas the increasing Nuc-tRFs shared only several of their pathways, possibly due to having the lowest number of predicted targets (Supplementary Figure 6c).

MT-Gly-i-tRF family correlates with maternal BMI

Searching whether the expression of certain tRF families correlated to participant characteristics such as gestation age, newborn weight etc., revealed a strong correlation between the MT-Gly-i-tRF family and maternal BMI. 40 of the 59 members in this family showed high positive correlation between the tRF expression levels and maternal BMI during the third trimester, and 35 also correlated with the mothers' body weight at the same time point (Supplementary Table 5). Interestingly, the interaction between Glycine (Gly) and weight has been shown previously, with circulating Gly showing an inverse correlation with metabolic disorders such as obesity, non-alcoholic fatty liver disease, and type 2 diabetes [17–19]. Moreover, Gly was shown to be important in late stages of pregnancy [20], and one of its related tRFs, the nuclear 5'-tRF-GlyGCC, was proposed as a regulator of obesity-associated

pathways in human breast cancer cell lines, as well as mice and pigs [21, 22], altogether suggesting that these findings were not coincidental.

Third trimester Fetal stress index (FSI) enhance classification results in male newborns

Interestingly, combining our results with the previously published FSI from the FELICITy cohort, a measurement of the fetus heart rate reactivity measured non-invasively during the third trimester [1](Supplementary Figure 8a) elevated some of the classification results (Supplementary Figure 8d-h, Supplementary Table 8, 9), although it was not correlated with any of the tRFs or miRs and by itself did not yield more than 48% AUC. This was especially the case in male newborns, where combination of the FSI and all four DE miRs from UCS and maternal serum achieved an AUC of 95% (FDR = 0.058; Supplementary Figure 8g), an elevation of approximately 60%. In females, CholinotRFs together with FSI had an increase of 5%, reaching an AUC of 100% (FDR = 0.008; Supplementary Figure 8d). A similar elevation was observed when separating stress and control groups of males and females together, with the combination of FSI and all DE tRFs yielding an AUC of 83% (FDR = 0.029, 5% elevation, Supplementary Figure 8e). Most of the other marker sets did not do so well with the addition of FSI, showing decrease in classification success (Supplementary Table 8, 9). Together, these findings indicate that newborns can be differentiated by their mothers' PPS already at birth, as reflected by UCS short noncoding RNAs, with the sex of the newborn playing a significant role in determining the type of markers required.

SUPPLEMENTARY FIGURES

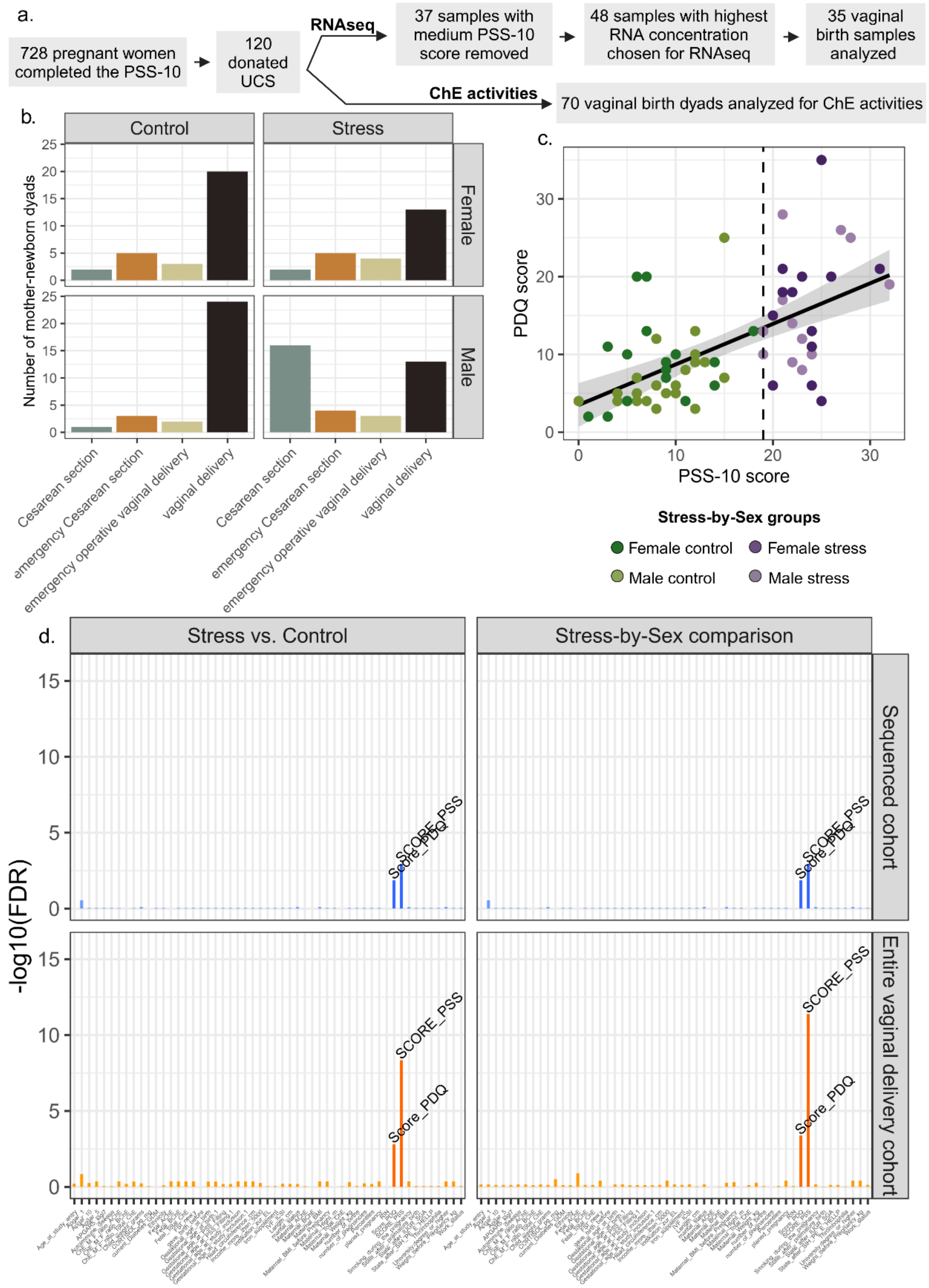

Supplementary Figure 1 - **Data characteristics.** (a) Of 728 pregnant women filled the Cohen PSS-10 during their third trimester, 120 gave UCS at birth. Next, only samples of dyads with either low (PSS-10 ≤ 10) or high (PSS-10 ≥ 19) PPS were considered for short RNA sequencing. In each of the four groups, the 12 samples with the highest concentration in both Nanodrop and Bioanalyzer assessment were chosen for sequencing (n=48). Post-sequencing, four samples were disqualified due to extremely low/high counts and finally only samples from

vaginal birth dyads were analyzed. ChEs activities were measured from all 70 vaginal birth dyads. (b) Bar plot showing the distribution of birth type in the four study groups, assigned according to the mothers' PPS by PSS-10 and the newborn sex. 58.3% of 128 mothers experienced normal vaginal delivery. (c) PSS-10 and PDQ show significant positive correlation across individuals (Pearson R = 0.6, P-value = 5.3e-08). (d) Bar plot presenting testing features across groups using Kruskal-Wallis test with FDR correction. Only the two stress-assessing questionnaires (PSS-10 and PDQ) were significantly different between the groups. Created with BioRender.

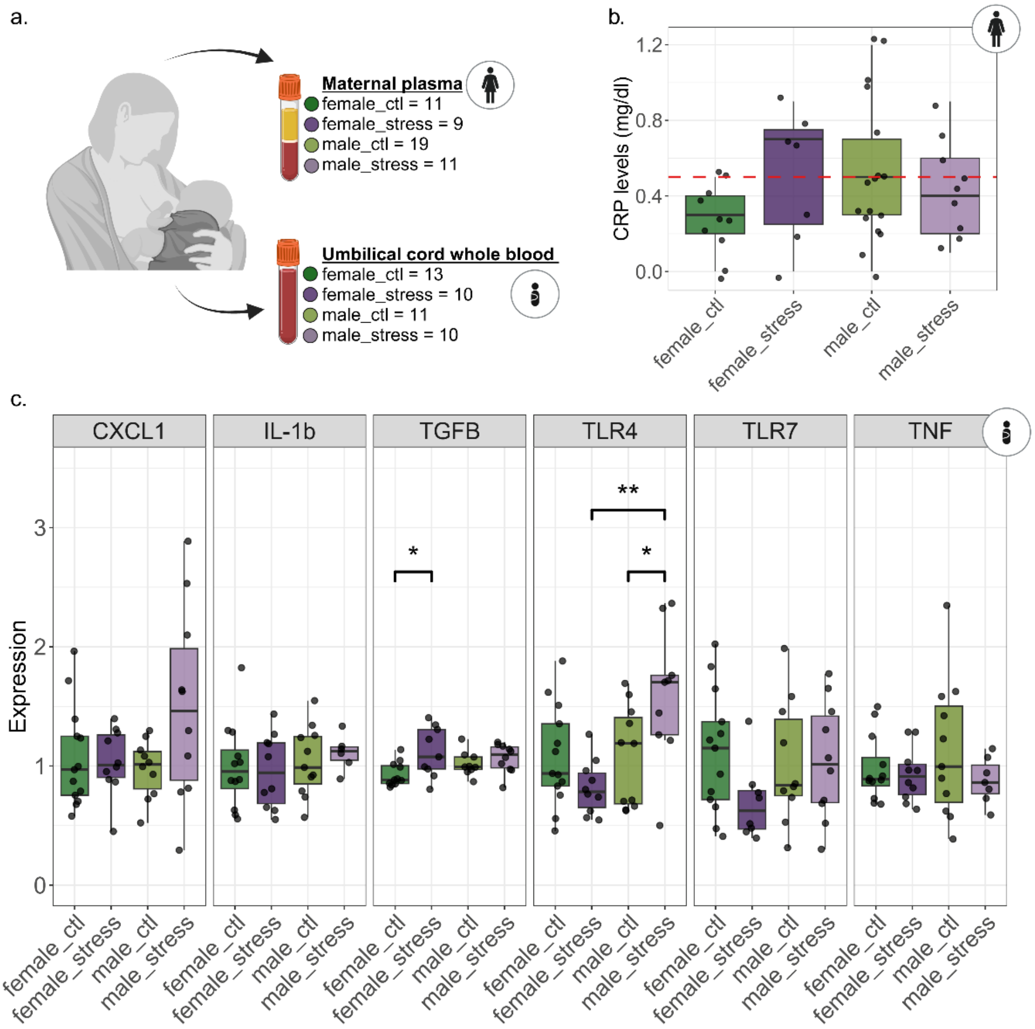

Supplementary Figure 2 – **Immune biomarkers show sex-specific effects in newborns.** (a) Illustration of the maternal CRP and the newborns umbilical cord whole blood cohorts. (b) Boxplots depicting maternal CRP levels across the four maternal groups; red line indicating the cutoff for infection-level CRP at 0.5 mg/dl. (c) Boxplots presenting RT-qPCR expression levels of six inflammatory biomarkers in DDCt, across the four newborn stress-by-sex groups. Wilcox test with significance set at p = 0.05. Created with BioRender.

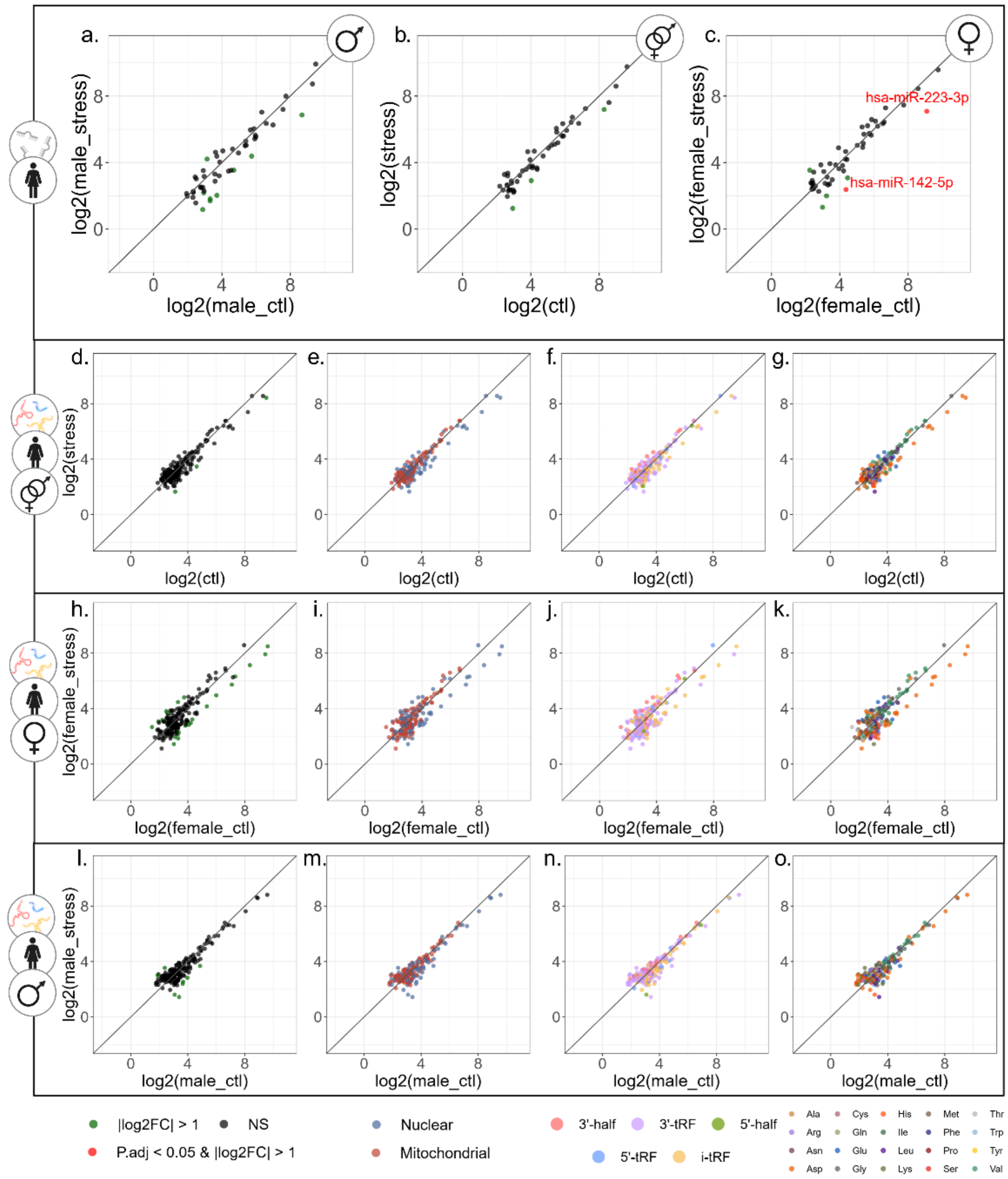

Supplementary Figure 3 - **Maternal tRF and miR profiles do not present similar effects to newborns.** Scatter plots of DE miRs (a-c) and tRFs (d-o), in (b, d-g) PPS vs. control mothers of male and female newborns (12 vs. 12), in (c, h-k) PPS vs. controls mothers of female newborns (6 vs. 6), and in (a, l-o) PPS vs. control mothers of male newborns (6 vs. 6). tRF scatter plots colored according to (e, i, m) genome origin, (f, j, n) tRF cleavage type, and (g, k, o) coded amino acid. Created with BioRender.

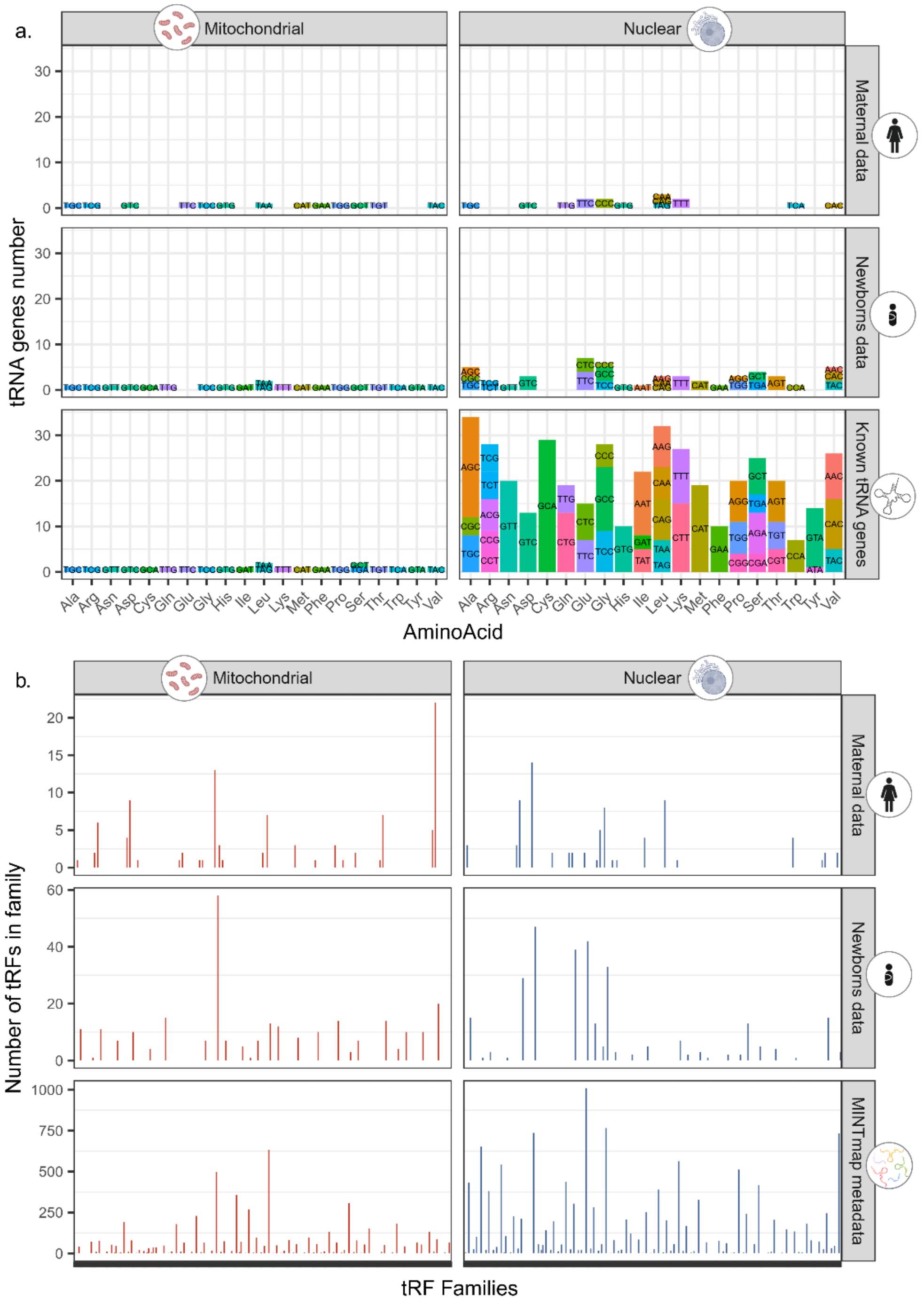

Supplementary Figure 4 - **tRNA genes and tRF families' distribution across data sets.** (a) Bar plot presenting the distribution of tRNA genes represented in our data compared to known tRNA genes. The left Y-axis shows the number of tRNA genes, on the right the division by cohort: mothers, newborns, and known tRNA genes by GtRNAdb for Nuc tRNA genes and mitotRNAdb for MT ones. The top X axis divides tRNA genes into MT and Nuc genome origin, and the bottom to the coded amino acid. The bar color depicts tRNA codons. (b) Bar plot presenting tRF families' size, grouped based on genome origin, cleavage type, and coded amino acid. The left Y-axis shows tRF numbers in the family, and the right division to cohorts: mothers, newborns, and all possible tRFs according to the combined metadata files by MINTmap. The top X axis shows division to MT and Nuc genome origin and the bottom to tRF families. Created with BioRender.

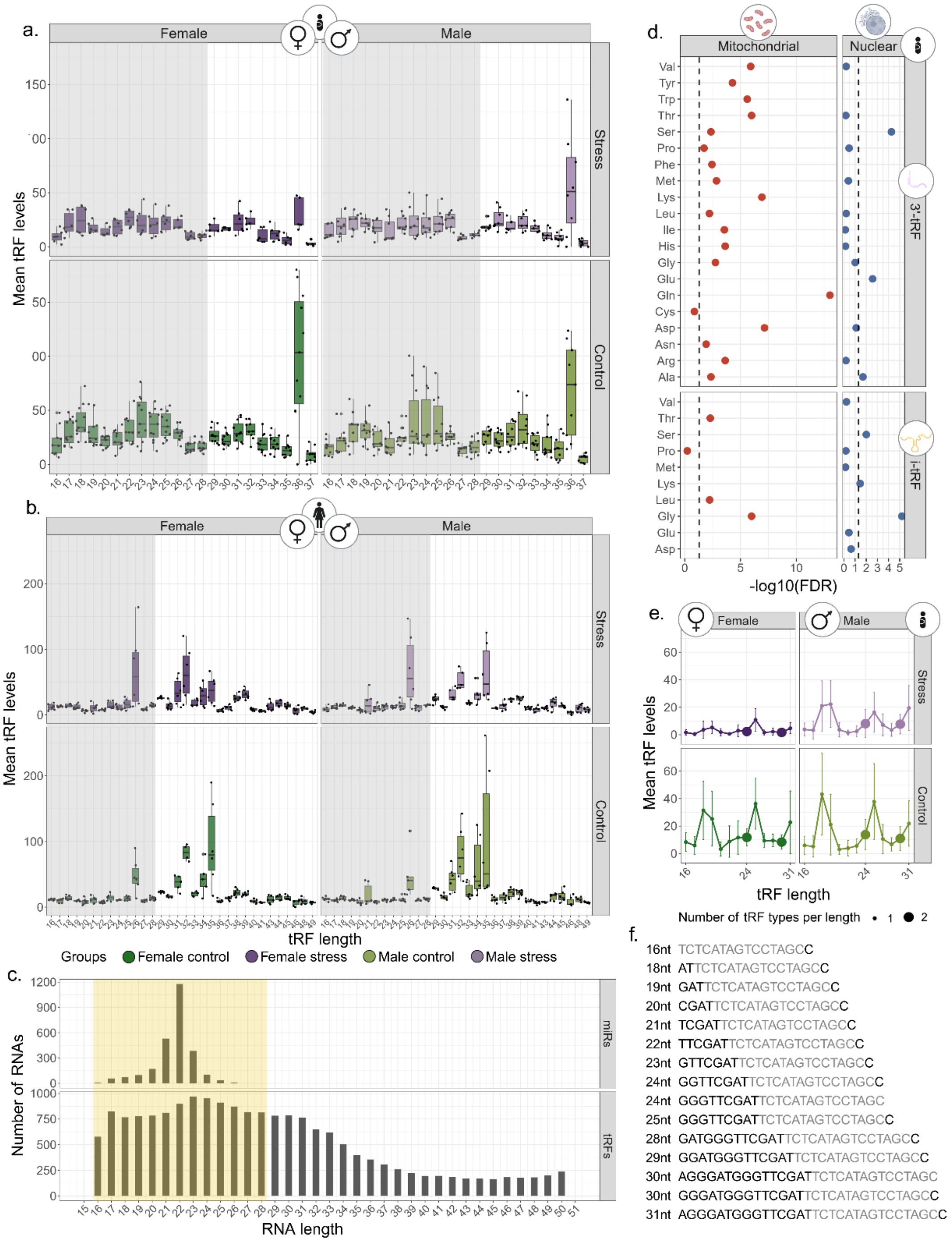

Supplementary Figure 5 - **tRF families differ in length across newborn stress and sex groups.** (a) Boxplot showing mean expression levels of all tRFs of each length, calculated separately for the newborns of the four stress-by-sex groups (n=35), with outliers identified and excluded from the analysis by the IQR method. The black dots represent mean values for single participants. The grey areas are lengths that overlap with miR length according to miRBase (16-28nt). (b) the same for the mothers (n=24). (c) Length distribution of miRs and tRFs in known data sets. The x-axis shows lengths in nucleotides, y-axis shows the number of RNAs. miRs length distribution is taken from miRbase, and tRFs length distribution is taken from MINTbase metadata. (d) Kruskal-Wallis (KW) test of length distributions between the four groups, for each tRF family, of nuclear and mitochondrial origin. The dashed line marks the threshold of significance (FDR = 0.05). (e) Dot plot of the KW test for length distribution of the top tRF family, MT-Gln-3'-tRF, in each of the four groups. The length of tRFs in the family is

plotted vs. their mean levels and the dot size reflects the number of tRFs sharing the same length in that family. (f) The sequences of all MT-Gln-3'-tRF family members in our data, from the shortest to the longest. Grey letters mark the sequence shared by all members. Created with BioRender.

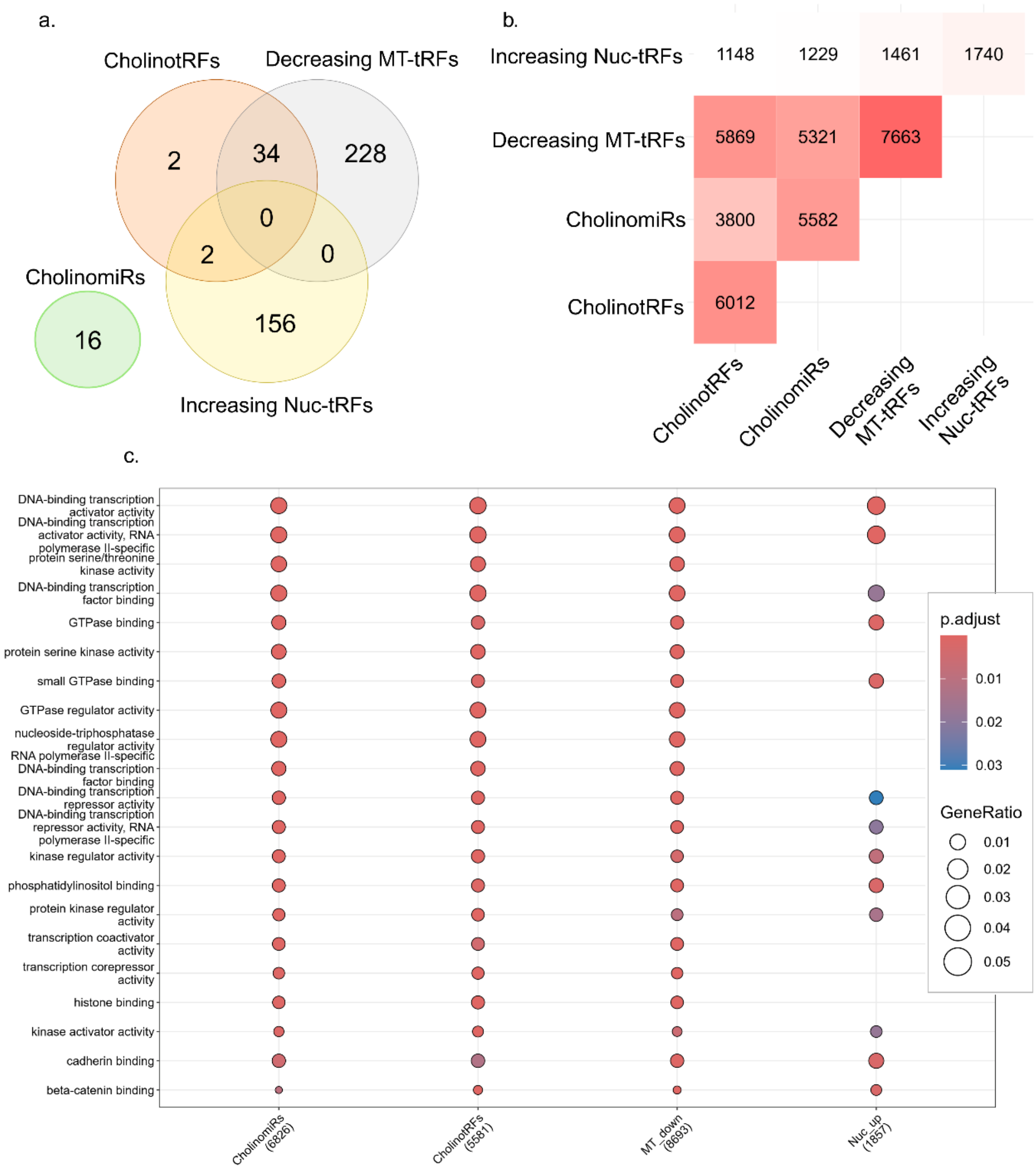

Supplementary Figure 6 – **Pathways of predicted targets of tRF groups present similar transcription-related origin.** (a) Venn plot showing overlap between the four groups of RNAs from the UCS were scanned for gene ontology (GO) analysis: (1) MT-tRFs decreasing in the stress groups, (2) Nuc-tRFs increasing in the stress groups, (3) CholinotRFs, and (4) CholinomiRs. (b) Heatmap showing overlap between predicted targets of the four marker groups. (c) GO plot showing top molecular function pathways based on said pathways and their gene ratio and adjusted p.value. Created with BioRender.

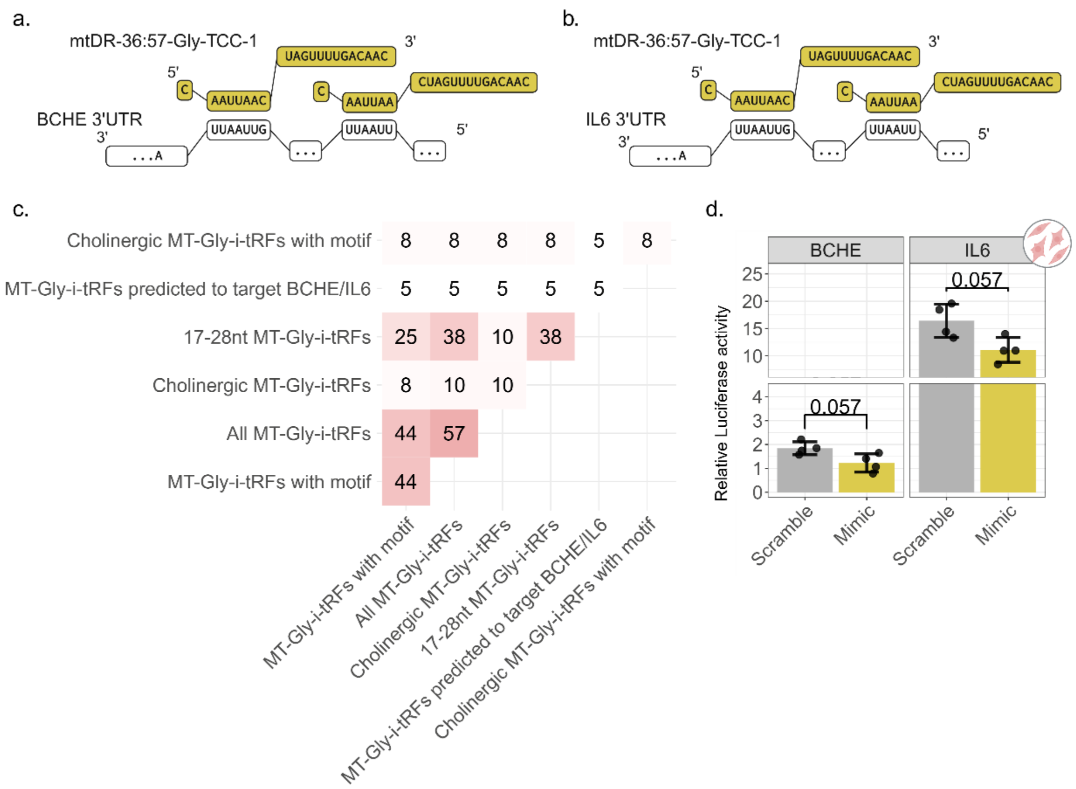

Supplementary Figure 7 – **Dual luciferase assay showing that tRF mtDR-36:57-Gly-TCC-1 reduces BCHE and IL6 levels. (**a) Illustration of possible seed interaction between tRF mtDR-36:57-Gly-TCC-1 and BCHE 3'UTR, as proposed by the miRDB algorithm. (b) the same for IL6 3'UTR. (c) Heatmap showing overlaps between the members of the MT-Gly-i-tRF family which may evoke the same interactions as mtDR-36:57-Gly-TCC-1 with BCHE and/or IL6. The "motif" is the sequence of tRF mtDR-36:57-Gly-TCC-1 - CAATTAACTAGTTTTGACAAC. (d) Luciferase activity in HEK293T cells co-transfected with a plasmid expressing human BCHE or IL6 3'UTR and tRF mtDR-36:57-Gly-TCC-1 (mimic) or its scrambled sequence (scramble). Bar graphs show average ± SD, P-value calculated by Wilcox test. Created with BioRender.

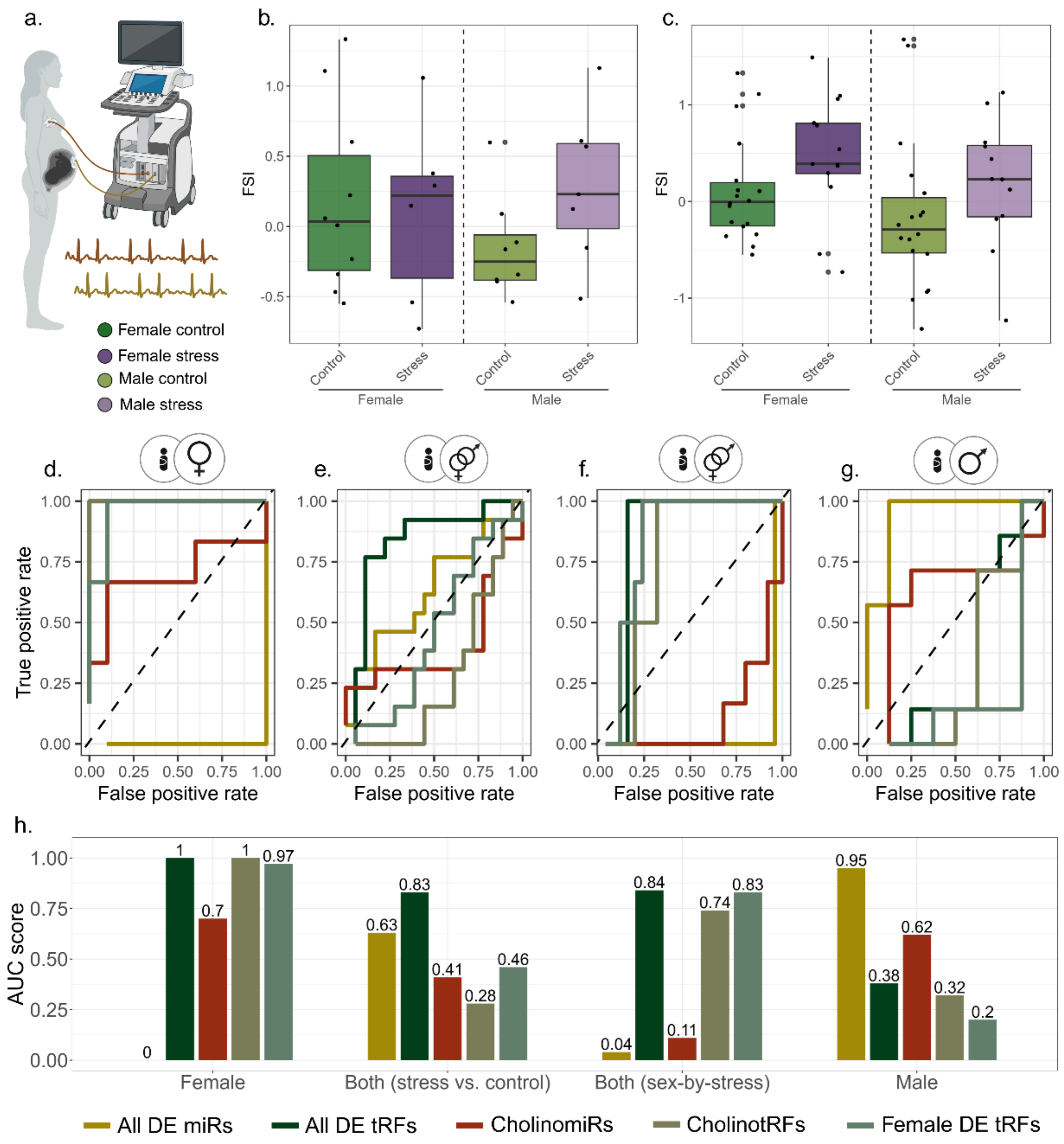

Supplementary Figure 8 - **FSI increases the accuracy of PPS classification of some of the marker groups.** (a) FSI measurement of the fetus's heart rate reactivity measured non-invasively during the third trimester. (b-c) Boxplots of FSI results, (b) in the sequenced cohort (n = 35 dyads), and (c) in the entire cohort of only vaginal deliveries (n = 70 dyads). None of the comparison was significant, although comparing female newborns stress and control dyads came close (u.test, P-value = 0.062). (d-g) ROC curves of five marker groups (All DE tRFs, Female DE tRFs, CholinotRFs, All DE miRs, and CholinomiRs), classifying newborns to mothers' PPS and control groups based on SVM Kernel algorithm with "leave one out" cross validation: (d) females (n= 6 vs. n=11), (e) male & female (stress vs. control; n= 14 vs. n=21), (f) male & female (stress-by-sex; n= 14 vs. n=21), and (g) males (n= 8 vs. n=10). (h) bar plot of AUC values across comparisons (FDR based on 10,000 permutations available at Supplementary Table 9). Created with BioRender.

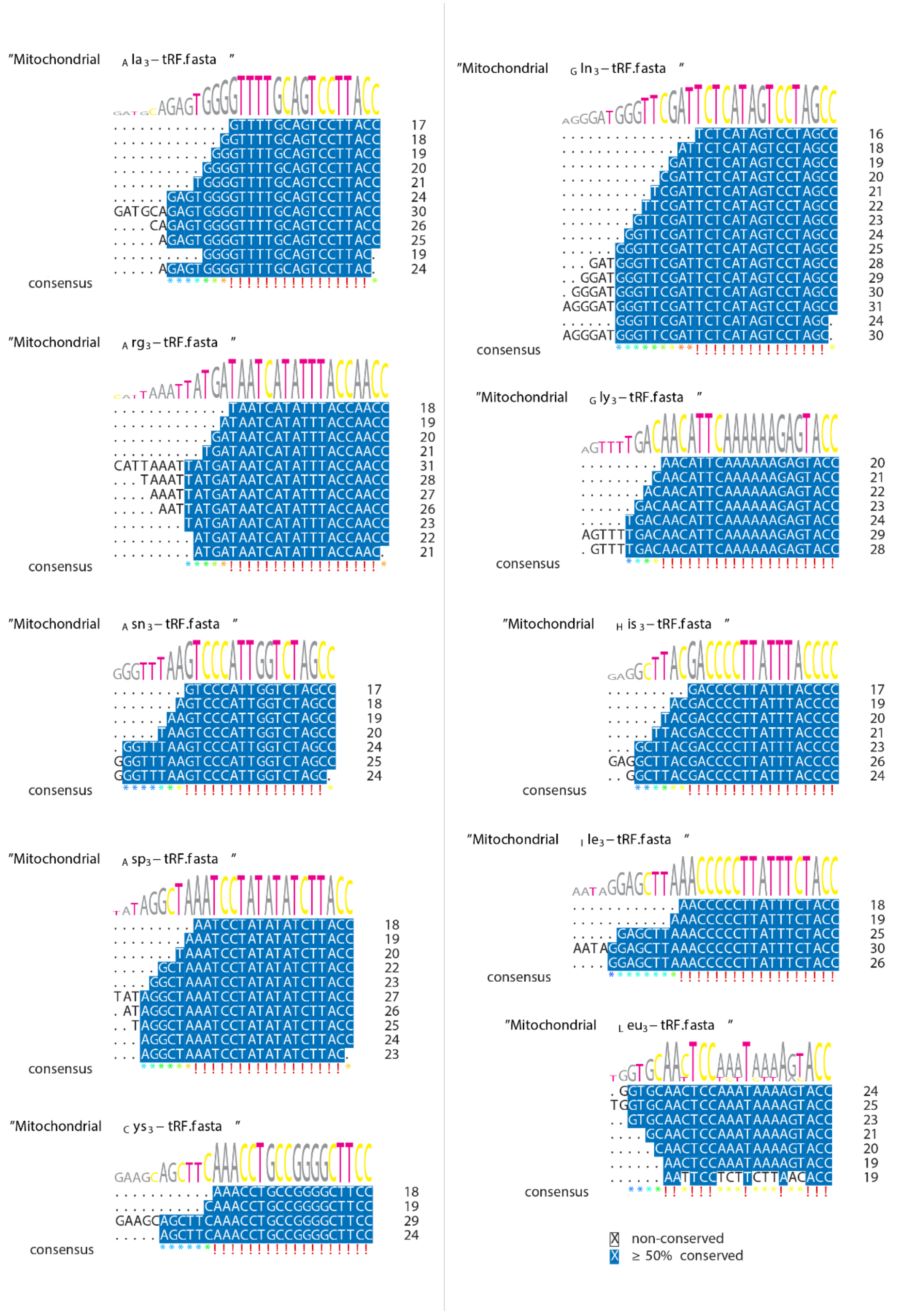

Supplementary Figure 9 – **tRF families share sequence similarities.** Each box shows the multiple sequence alignment (MSA) of all the tRF members in our data that belong to the same tRF family. Each line shows the relevant sequence of a specific tRF in the family, with the numbers on the right side indicating the length of each tRF. White background shows non-conserved nucleotides and blue shows conservation in more than 50% of sequences. The

consensus sequence is shown on top of each box. A total of 48 families are depicted over pages 10-15.

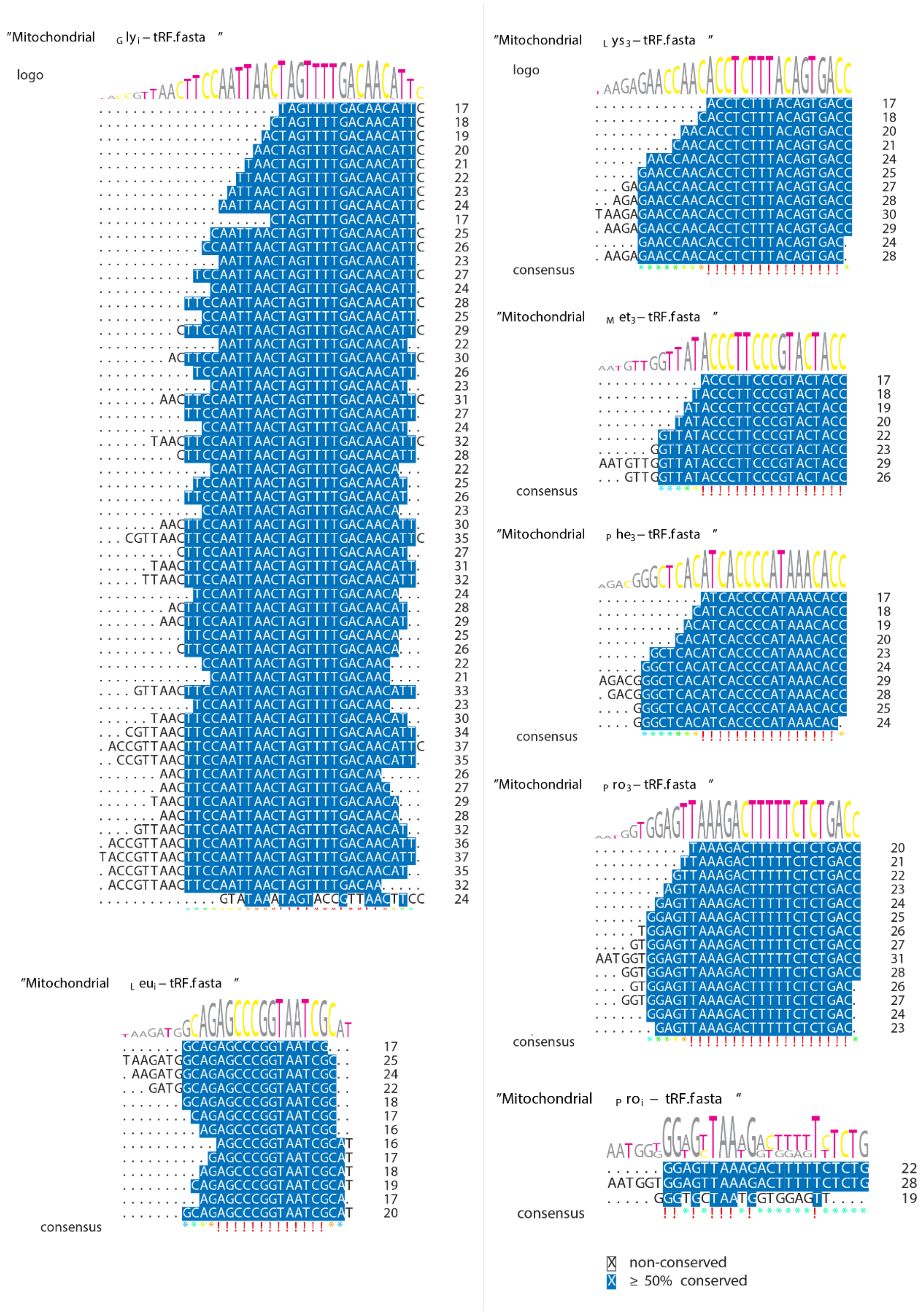

Supplementary Figure 9 – **continue.**

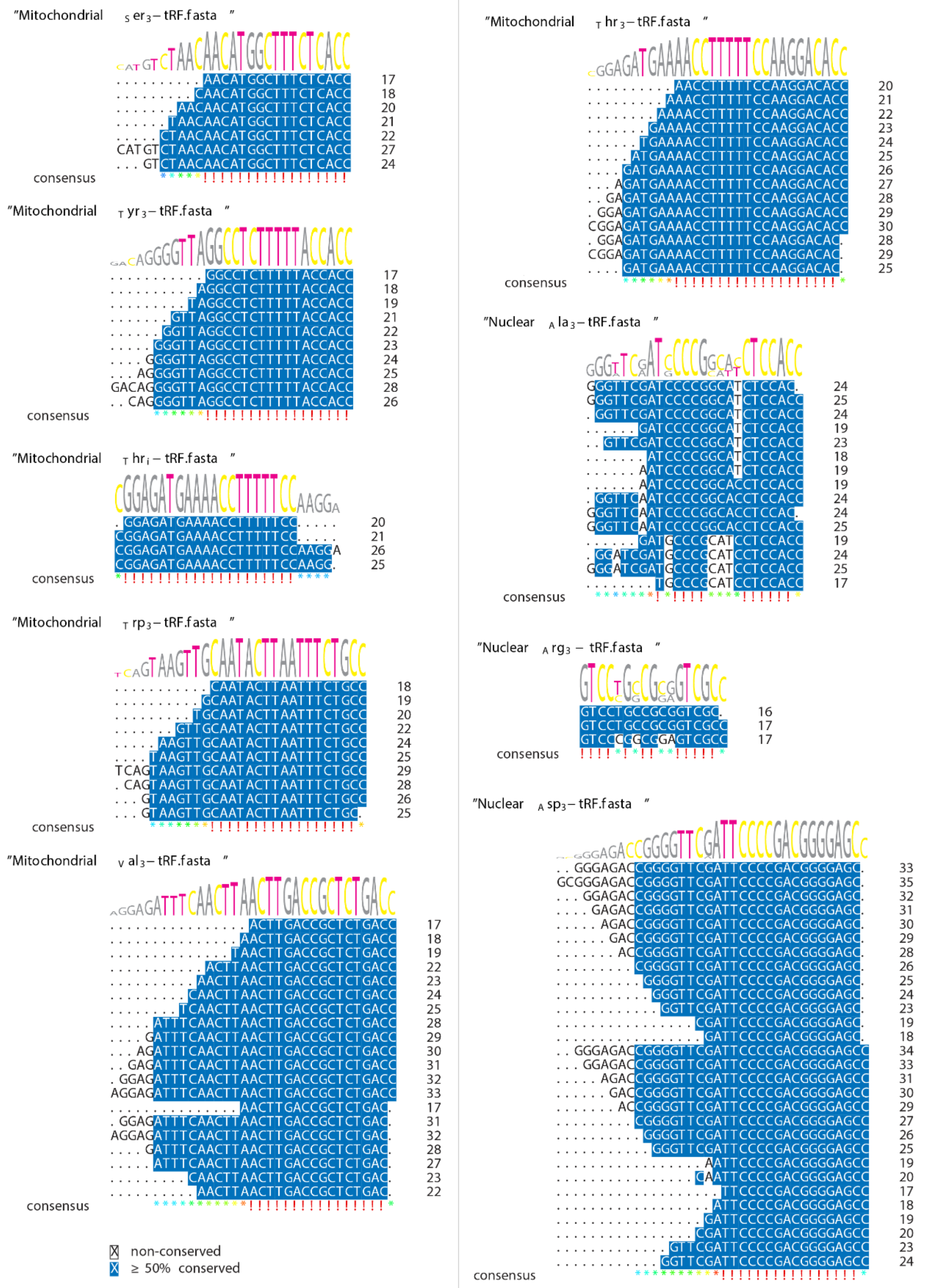

Supplementary Figure 9 – **continue.**

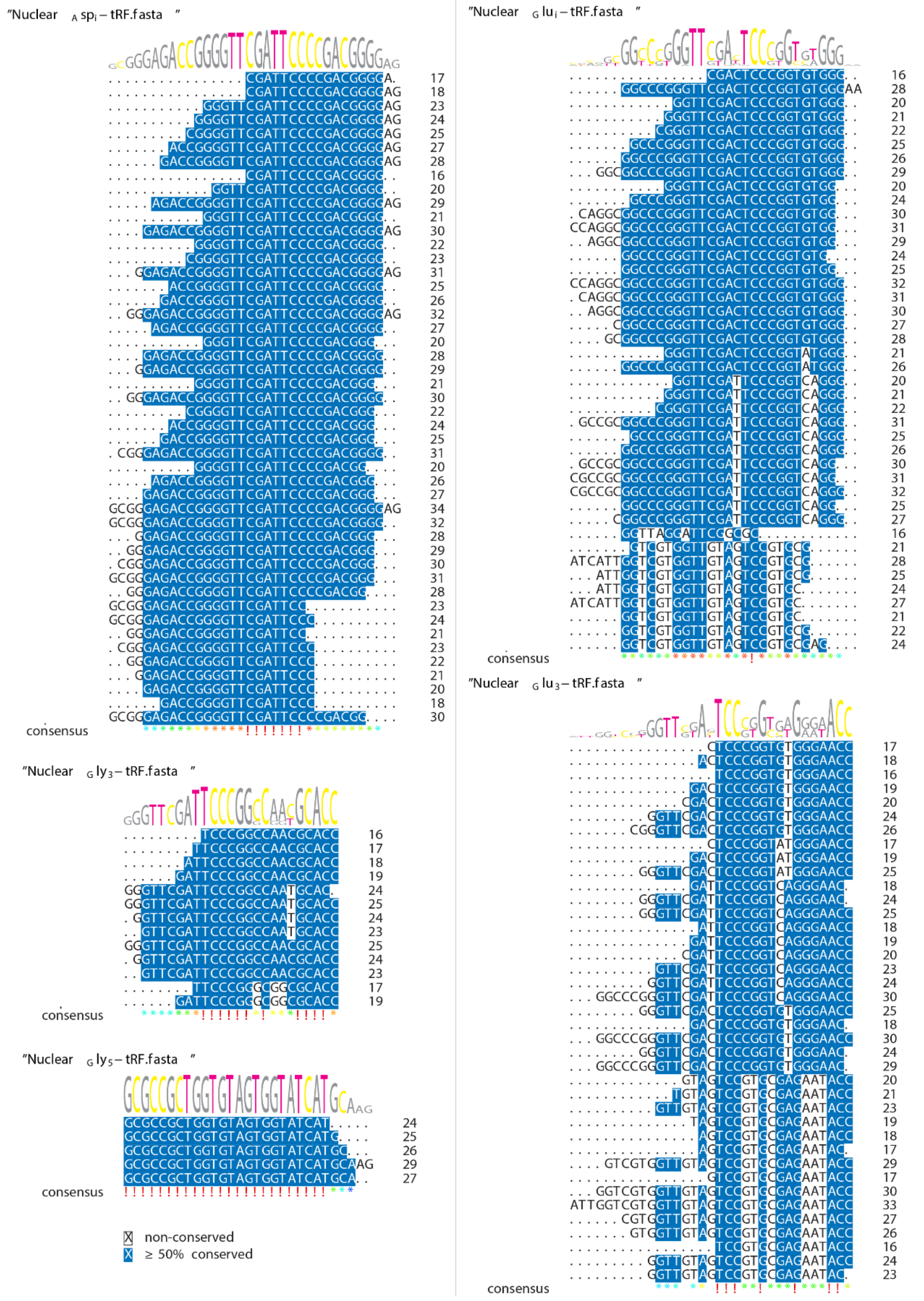

Supplementary Figure 9 – **continue.**

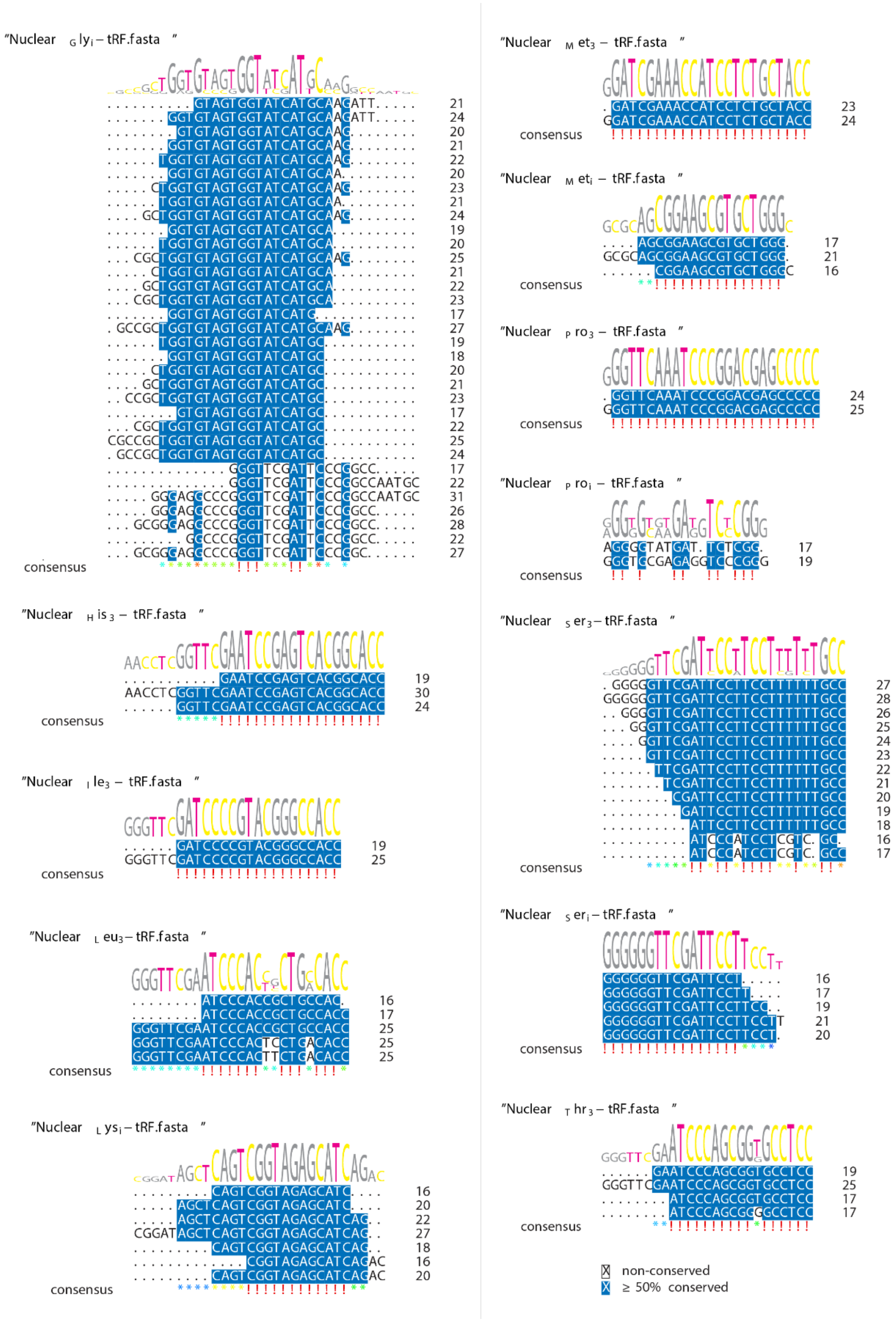

Supplementary Figure 9 – **continue.**

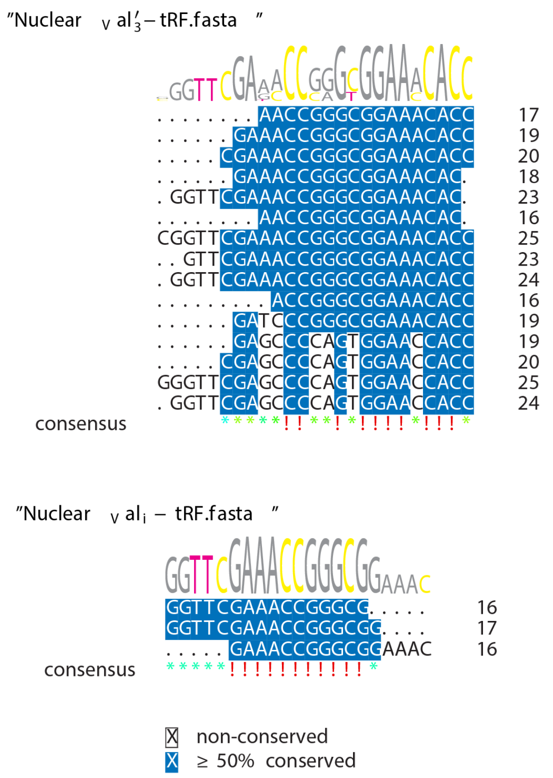

Supplementary Figure 9 – **continue.**

SUPPLEMENTARY REFERENCES

1. Lobmaier SM, Müller A, Zelgert C, Shen C, Su PC, Schmidt G, et al. Fetal heart rate variability responsiveness to maternal stress, non-invasively detected from maternal transabdominal ECG. Arch Gynecol Obstet. 2020;301:405–414.

2. Kilpatrick SK, Ecker JL. Severe maternal morbidity: screening and review. Am J Obstet Gynecol. 2016;215:B17–B22.

3. Gordijn SJ, Beune IM, Thilaganathan B, Papageorghiou A, Baschat AA, Baker PN, et al. Consensus definition of fetal growth restriction: a Delphi procedure. Ultrasound in Obstetrics & Gynecology. 2016;48:333–339.

4. Ellman GL, Courtney KD, Andres V, Featherstone RM. A new and rapid colorimetric determination of acetylcholinesterase activity. Biochem Pharmacol. 1961;7:88–95.

5. Wille T, Thiermann H, Worek F. Evaluation of 6,6′-dithionicotinic acid as alternative chromogen in a modified Ellman method—comparison in various species. Toxicol Mech Methods. 2011;21:533–537.

6. R Core Team. R: A Language and Environment for Statistical Computing. 2019. 2019.

7. Magee R, Rigoutsos I. On the expanding roles of tRNA fragments in modulating cell behavior. Nucleic Acids Res. 2020;48:9433–9448.

8. Pliatsika V, Loher P, Telonis AG, Rigoutsos I. MINTbase: A framework for the interactive exploration of mitochondrial and nuclear tRNA fragments. Bioinformatics. 2016;32:2481–2489.

9. Bodenhofer U, Bonatesta E, Horejš-Kainrath C, Hochreiter S. msa: an R package for multiple sequence alignment. Bioinformatics. 2015;31:3997–3999.

10. Telonis AG, Kirino Y, Rigoutsos I. Mitochondrial tRNA-lookalikes in nuclear chromosomes: Could they be functional? RNA Biol. 2015;12:375–380.

11. Suzuki T, Yashiro Y, Kikuchi I, Ishigami Y, Saito H, Matsuzawa I, et al. Complete chemical structures of human mitochondrial tRNAs. Nat Commun. 2020;11.

12. Loher P, Telonis AG, Rigoutsos I. MINTmap: fast and exhaustive profiling of nuclear and mitochondrial tRNA fragments from short RNA-seq data. Sci Rep. 2017;7:41184.

13. Holmes AD, Chan PP, Chen Q, Ivanov P, Drouard L, Polacek N, et al. A standardized ontology for naming tRNA-derived RNAs based on molecular origin. Nat Methods. 2023;20:627–628.

14. Vaknine S, Soreq H. Central and peripheral anti-inflammatory effects of acetylcholinesterase inhibitors. Neuropharmacology. 2020;168:108020.

15. Baines KJ, West RC. Sex differences in innate and adaptive immunity impact fetal, placental, and maternal health. Biol Reprod. 2023;109:256–270.

16. Wu T, Hu E, Xu S, Chen M, Guo P, Dai Z, et al. clusterProfiler 4.0: A universal enrichment tool for interpreting omics data. Innovation. 2021;2.

17. Tan HC, Hsu JW, Tai ES, Chacko S, Wu V, Lee CF, et al. De Novo Glycine Synthesis Is Reduced in Adults With Morbid Obesity and Increases Following Bariatric Surgery. Front Endocrinol (Lausanne). 2022;13.

18. Alves A, Bassot A, Bulteau AL, Pirola L, Morio B. Glycine metabolism and its alterations in obesity and metabolic diseases. Nutrients. 2019;11.

19. Imenshahidi M, Hossenzadeh H. Effects of glycine on metabolic syndrome components: a review. J Endocrinol Invest. 2022;45:927–939.

20. Rasmussen BF, Ennis MA, Dyer RA, Lim K, Elango R. Glycine, a Dispensable Amino Acid, Is Conditionally Indispensable in Late Stages of Human Pregnancy. Journal of Nutrition. 2021;151:361–369.

21. Chen F, Song C, Meng F, Zhu Y, Chen X, Fang X, et al. 5’-tRF-GlyGCC promotes breast cancer metastasis by increasing fat mass and obesity-associated protein demethylase activity. Int J Biol Macromol. 2023;226:397–409.

22. Liao T, Gan M, Lei Y, Wang Y, Chen L, Shen L, et al. Dynamic changes in the transcriptome of tRNA-derived small RNAs related with fat metabolism. Sci Data. 2023;10.
